# Efficacy of High-intensity Statin Use for Transient Ischemic Attack Patients with Positive Diffusion-weighted Imaging

**DOI:** 10.1038/s41598-018-36986-w

**Published:** 2019-02-04

**Authors:** Bo Song, Yuan Cao, Lulu Pei, Hui Fang, Lu Zhao, Pei Chen, Pan Si, Xinjing Liu, Kai Liu, Yuan Gao, Jun Wu, Shilei Sun, Xiaoying Wang, Eng H. Lo, Ferdinando S. Buonanno, Mingming Ning, Yuming Xu

**Affiliations:** 1 Department of Neurology, The First Affiliated Hospital of Zhengzhou University, Zhengzhou, Henan China; 2 The Departments of Radiology and Neurology, Massachusetts General Hospital and Harvard Medical School, Boston, MA USA; 3 Clinical Proteomics Research Center and Cardio-Neurology Clinic, Department of Neurology, Massachusetts General Hospital, Harvard Medical School, Boston, MA USA

## Abstract

To determine whether positive or negative DWI TIA patients could get benefits from HST we conducted a cohort study which data from the prospective, hospital-based, TIA database of the First Affiliated Hospital of Zhengzhou University. The end-point was 7-day and 90-day incidence of stroke. Cox proportional hazard regression models were used to analyze the association between end-points and high-intensity statin treatment in TIA patients with positive and negative DWI. A total of 987 eligible TIA patients were analyzed. The stroke risk of patients with positive DWI was about a four-fold increase compared to that with negative DWI (7 d, 10.9 versus 1.8, p < 0.001 and 90 d, 18.3 versus 4.2, p < 0.001). After adjusting confounding factors, HST significantly improved both 7-day (HR 0.331, 95% CI 0.165–0.663; p = 0.002) and 90-day (HR 0.480, 95% CI 0.288–0.799; p = 0.005) outcomes in positive DWI patients. As a conclusion, high-intensity statin use reduces the 90 days’ recurrent stroke risk in DWI-positive TIA patients but not in DWI-negative patients.

## Introduction

Transient ischemic attack (TIA) patients with positive diffusion-weighted imaging (DWI) are at a high risk of early stroke^1,2^. Following many changes in the management of TIA, recent studies reported that each additional infarct on brain imaging of post TIA patients doubled the risk of stroke^3^. Therefore, aggressive medication therapy is essential for those TIA patients at high risk.

Statin is the recommended lipid-lowering agent of atherosclerotic stroke and TIA patients. It is uncertain whether DWI-positive TIA patients, which may be a different population compared to atherosclerotic TIA, could benefit from high-intensity statin therapy (HST). In addition, long term high-intensity statin therapy could bring about not only a heavy economic burden but also potential adverse events for those patients. As a result, to give a precise therapy for certain individuals is necessary. The present study aimed to investigate whether HST could improve clinical outcomes of TIA patients using imaging criterion such as DWI positivity.

## Methods

The TIA database of the First Affiliated Hospital of Zhengzhou University prospectively enrolled consecutive hospitalized patients with a diagnosis of TIA (within 7 days of ictus) since 2010. This study was approved by the Ethics Committee of the First Affiliated Hospital of Zhengzhou University. All methods applied in our study were performed in accordance with relevant guidelines^4^. All patients or their legally authorized representatives signed an informed consent form. The datasets analyzed during the current study are available from the corresponding author on reasonable request.

### Study Population

TIA was diagnosed based on World Health Organization (WHO) diagnostic criteria, which defined a TIA as an acute loss of focal cerebral or ocular function lasting less than 24 h attributed to embolic or thrombotic vascular diseases^5^. Stroke was defined as the sudden onset of neurological symptoms that persisted for ≥24 h^5^. Those excluded were patients who did not have MRI within 7 days of ictus, refused to participate or did not complete the follow-up protocol or had severe disorders such as cancer and hepatic disease.

Trained physicians recorded all information of patients by using paper case report forms. Acute positive DWI was defined as lesion(s) consistent with acute cerebral ischemia, as determined by the neuroradiologist and stroke physician blinded to patient outcome. Atherosclerotic TIA patients were defined to have an LDL cholesterol level of at least 100 mg per deciliter (2.6 mmol per liter) and no more than 190 mg per deciliter (4.9 mmol per liter)^6^.

According to 2013 ACC/AHA guideline^4^, atorvastatin 40 to 80 mg and rosuvastatin 20 to 40 mg are referred to as HST. HST group was defined as TIA patients with high-intensity statin therapy during hospitalization before stroke or any mortality, and the rest were identified as non-high-intensity statin therapy (non-HST) group.

Patients enrolled in the database were followed up by telephone interview. All patients suspected of stroke were followed up via face-to-face interview to insure they fit strict TIA criterion.

### Statistical Analyses

Patients were divided into positive DWI group and negative DWI group according to whether they had acute positive DWI lesions. For baseline clinical and imaging characteristics, categorical variables were analyzed by χ2 test and continuous variables were compared using t-test. Subsequently, cumulative event rates were estimated with the Kaplan–Meier method, and differences were tested with a log-rank test. Then, Cox proportional hazard regression models were used to explore the association between the survival of patients and clinical factors. Associations were presented as hazard ratio (HR) with corresponding 95% confidence interval (CI). All reported probability values are for 2-sided tests with a pre-specified α of 0.05. Statistical analysis was performed using IBM SPSS Statistics version 19.0 (SPSS, Inc., Chicago, IL, USA) and STATA version 12.0.

## Results

From October 2010 to March 2016, a total of 1087 eligible TIA patients were enrolled and excluding 17 patients lost to followup and 83 patients without DWI image. Comparison of the baseline characteristics of the included and excluded data showed no significant difference. As a result, 987 eligible TIA patients were analyzed. Of the included patients, the average age was 56.90 ± 12.64 years, and 378 patients (38.3%) were female.

According to whether they had acute positive DWI lesions, patients were categorized into positive DWI group and negative DWI group. There were 387 patients with positive DWI, 48.1% were treated with HST, 53 patients (5.4%) experienced a stroke within 7 days and 96 patients (9.7%) within 90 days. The stroke risk of patients with positive DWI was about a four-fold increase compared to that with negative DWI (7 d, 10.9 versus 1.8, p < 0.001 and 90 d, 18.3 versus 4.2, p < 0.001) (Table 1). The number of patients with stroke history and with ABCD2 ≥ 4 in positive DWI group were higher than that in negative DWI group (23.8 vs. 18.2, *p* = 0.035; 49.4 vs. 35.0, *p* < 0.001). In addition, the rate of HST and dual antiplatelet therapy in positive DWI group were also significant higher than that in negative DWI group (53.7 vs. 44.5, *p* = 0.005; 49.6 vs. 42.0, *p* = 0.022).

**Table 1:** Baseline characteristics of TIA patients included in the study stratified by DWI.

Variables	Positive DWI n = 387 (39.2%)	Negative DWI n = 600 (60.8%)	p value
Age, mean ± SD, years	56.86 ± 12.97	56.93 ± 12.44	0.934
Gender (Female), n (%)	35.4	40.2	0.140
Smoking, n (%)	31.0	26.7	0.148
History of DM, n (%)	16.5	15.0	0.530
History of Hypertension, n (%)	57.6	53.2	0.190
History of Hyperlipemia, n (%)	18.3	16.7	0.492
History of Stroke, n (%)	23.8	18.2	0.035
History of AF, n (%)	2.3	2.1	0.866
TG, mean ± SD	1.44 ± 0.98	1.52 ± 1.14	0.251
TC, mean ± SD	3.90 ± 1.45	3.82 ± 1.49	0.369
HDL-C, mean ± SD	1.05 ± 0.41	1.03 ± 0.42	0.573
LDL-C, mean ± SD	2.39 ± 1.08	2.31 ± 1.08	0.216
Atherosclerotic TIA, n (%)	40.3	39.7	0.842
ABCD2 ≥ 4, n (%)	49.4	35.0	<0.001
**Medications in-hospital**			
Dual Antiplatelet Therapy, n (%)	49.6	42.0	0.022
HST, n (%)	53.7	44.5	0.005
Anti-hypertension Therapy, n (%)	35.7	37.3	0.636
Anti-diabetes Therapy, n (%)	17.3	14.3	0.209
Anticoagulant Therapy, n (%)	4.5	2.7	0.175
**Stroke risk**			
7 days, n (%)	10.9	1.8	<0.001
90 days, n (%)	18.3	4.2	<0.001

DWI: diffusion weighted imaging; Smoking: current or previous smoking; DM: diabetes mellitus; AF: atrial fibrillation; TG: triglycerides; TC: total cholesterol; HDL-C: high-density lipoprotein cholesterol; LDL-C: low-density lipoprotein cholesterol; HST: high-intensity statin therapy.

After adjusting confounding factors (including gender, smoking history, hypertension history, hyperlipemia history, ABCD2 ≥ 4 and hospitalized therapy), HST significantly improved both 7-day (HR 0.331, 95% CI 0.165–0.663; p = 0.002) and 90-day (HR 0.480, 95% CI 0.288–0.799; p = 0.005) outcomes in positive DWI patients. However, hypertension history and ABCD^2^ ≥ 4 were independently related to recurrent ischaemic stroke risk at 90 days (HR 2.098, 95% CI 1.232–3.573, *p* = 0.006; HR 1.836, 95% CI 1.129–2.985, *p* = 0.014). For negative DWI patients, the results were not statistically significant (7 d, HR 0.750, 95% CI 0.198~2.845; p = 0.672 and 90 d, HR 1.499, 95% CI 0.639~3.515; p = 0.352) (Table 2).

**Table 2:** Significant Predictors of Clinical Outcome at 7 days and 90 days.

Variables	p value	95% CI
**Positive DWI, n** = **387 (39.2%)**		
7 days		
Gender	0.989	0.995 (0.532–1.863)
Smoking	0.343	0.706 (0.343–1.450)
Hypertension history	0.051	2.005 (0.998–4.026)
Hyperlipemia history	0.593	1.261 (0.539–2.953)
ABCD2 ≥ 4	0.008	2.413 (1.256–4.638)
Anti-hypertension therapy	0.131	1.610 (0.868–2.987)
Anti-diabetes therapy	0.910	0.953 (0.417–2.180)
Dual antiplatelet therapy	0.436	0.776 (0.409–1.471)
HST	0.002	0.331 (0.165–0.663)
90 days		
Gender	0.464	1.196 (0.741–1.929)
Smoking	0.194	0.694 (0.400–1.204)
Hypertension history	0.006	2.098 (1.232–3.573)
Hyperlipemia history	0.634	1.171 (0.612–2.240)
ABCD2 ≥ 4	0.014	1.836 (1.129–2.985)
Anti-hypertension therapy	0.174	1.394 (0.863–2.252)
Anti-diabetes therapy	0.951	1.020 (0.544–1.913)
Dual antiplatelet therapy	0.095	0.656 (0.400–1.077)
HST	0.005	0.480 (0.288–0.799)
**Negative DWI, n** = **600 (60.8%)**		
7 days		
Gender	0.323	0.508 (0.133–1.945)
Smoking	0.427	0.532 (0.112–2.528)
Hypertension history	0.594	0.713 (0.206–2.474)
Hyperlipemia history	0.344	1.932 (0.495–7.546)
ABCD2 ≥ 4	0.433	1.637 (0.477–5.615)
Anti-hypertension therapy	0.214	0.375 (0.080–1.763)
Anti-diabetes therapy	0.654	0.621 (0.077–4.979)
Dual antiplatelet therapy	0.362	0.517 (0.125–2.133)
HST	0.672	0.750 (0.198–2.845)
90 days		
Gender	0.340	0.660 (0.281–1.550)
Smoking	0.743	0.855 (0.334–2.186)
Hypertension history	0.215	1.739 (0.725–4.167)
Hyperlipemia history	0.641	0.772 (0.260–2.290)
ABCD2 ≥ 4	0.159	1.793 (0.796–4.040)
Anti-hypertension therapy	0.186	0.534 (0.211–1.354)
Anti-diabetes therapy	0.425	0.552 (0.129–2.374)
Dual antiplatelet therapy	0.595	0.795 (0.342–1.852)
HST	0.352	1.499 (0.639–3.515)

DWI: diffusion weighted imaging; AF: atrial fibrillation; HST: high-intensity statin therapy.

**Figure 1:**
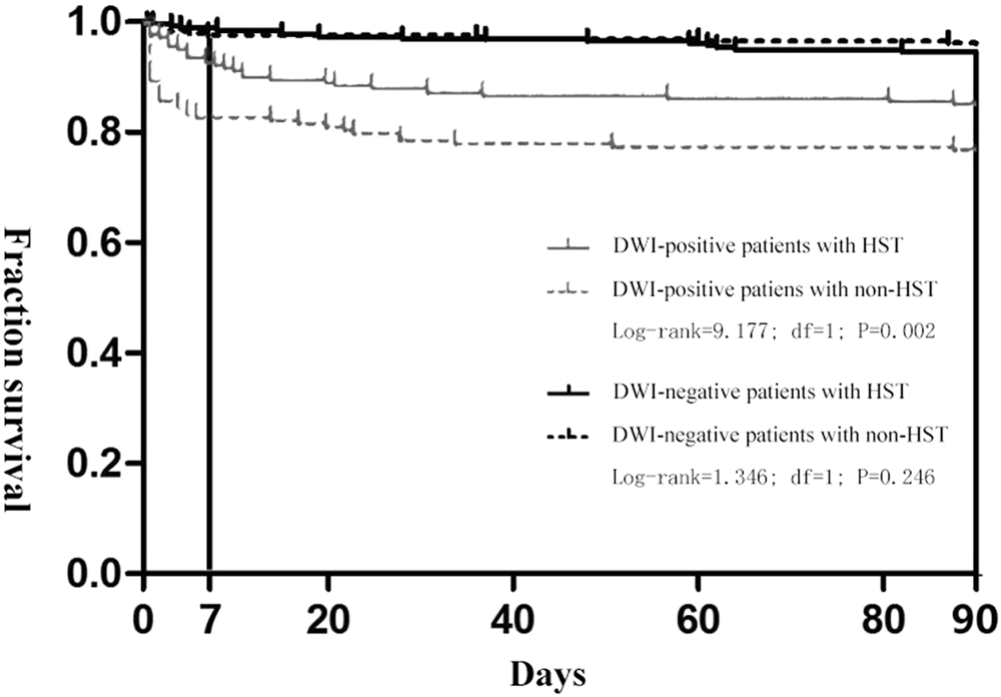
Early survival probability after TIA in patients with positive or negative DWI stratified by statin dosage during hospitalization at 90 days. High-intensity statin therapy could decrease 90-day stroke risk of DWI-positive TIA patients (log-rank = 9.177, *p* = 0.002). On the contrary, the stroke risk showed no significant difference between HST and non-HST groups in the DWI-negative TIA patients (log-rank = 1.346, *p* = 0.246).

The Kaplan–Meier indicated that HST could decrease 90-day stroke risk of positive DWI TIA patients (log-rank = 0.002). However, this effect did not remain significant among the negative DWI TIA patients (log-rank = 0.246) (Fig. 1).

## Discussion

Our study supported prior finding that early stroke risk among DWI-positive TIA patients was higher compared to that of negative DWI, as reported before^2,7^. We advance these finding in reporting new data from a prospective TIA cohort that there is a substantial reduction in early stroke risk in patients with DWI-positive TIA on high-intensity statin therapy during hospitalization.

The study presented a novel and fast approach to identifying target population of HST regardless of stroke subtype. DWI imaging, irrespective of etiology, is an easier and quicker way to get meaningful information before any further workup can be completed. Prior studies showed that TIA patients with positive DWI appeared to have more unstable phenotype and was associated with the greatest risk for imminent stroke, implying that an underlying stroke mechanism was active^3,8^. In our study, the percentages of atherosclerotic TIA in patients with DWI positive and negative imaging showed no significant difference. Hence, we speculate that the underlying mechanism of reduced stroke risk with early HST may not be only lipid-lowering, but also activation of other pleiotrophic effects of statin such as modification of the inflammatory cascade, antioxidant effects, upregulation of nitric oxide synthase with consequent increase in cerebral blood flow plaque stabilization, and modulating coagulation and platelet function^9^.

The results of our study may have importance clinically for TIA patients during acute hospitalization as the efficacy, safety, and cost-effectiveness must be balances when using HST. Urgent MRI DWI imaging maybe a rapid way to identify target population which may benefit the most from HST.

However, as an observational study, the selection bias could not be avoided and we could not draw conclusions on causality. For example, DWI positive patients had increased trend of being on dual-antiplatelet agents and being more aggressively treated with HST. There are also more stroke patients in this cohort. It is possible that DWI positive group may have more risk factors. We attempted to account for these important variables by adjusting for multiple confounders including gender, smoking history, stroke history, atrial fibrillation history, atherosclerotic TIA and hospitalized therapy and the results remain statistically significant. However, we acknowledge that this is a proof of principle study in a cohort of prospectively followed patient during acute hospitalization with limitation on generalizability. Further prospective randomized clinical trials are needed to validate these findings.
